# Changes of O^6^-Methylguanine DNA Methyltransferase (MGMT) Promoter Methylation in Glioblastoma Relapse—A Meta-Analysis Type Literature Review

**DOI:** 10.3390/cancers11121837

**Published:** 2019-11-21

**Authors:** Jonas Feldheim, Almuth F. Kessler, Camelia M. Monoranu, Ralf-Ingo Ernestus, Mario Löhr, Carsten Hagemann

**Affiliations:** 1 Tumorbiology Laboratory, Department of Neurosurgery, University of Würzburg, Josef-Schneider-Str. 11, D-97080 Würzburg, Germany; jonas.feldheim@googlemail.com (J.F.); Kessler_A1@ukw.de (A.F.K.); Ernestus_R@ukw.de (R.-I.E.); Loehr_M1@ukw.de (M.L.); 2 Department of Neuropathology, Institute of Pathology, University of Würzburg, Josef-Schneider-Str. 2, D-97080 Würzburg, Germany; camelia-maria.monoranu@mail.uni-wuerzburg.de

**Keywords:** glioblastoma multiforme (GBM), glioma, relapse, temozolomide, MGMT promoter methylation, therapy, resistance, recurrence

## Abstract

Methylation of the O^6^-methylguanine DNA methyltransferase (MGMT) promoter has emerged as strong prognostic factor in the therapy of glioblastoma multiforme. It is associated with an improved response to chemotherapy with temozolomide and longer overall survival. MGMT promoter methylation has implications for the clinical course of patients. In recent years, there have been observations of patients changing their MGMT promoter methylation from primary tumor to relapse. Still, data on this topic are scarce. Studies often consist of only few patients and provide rather contrasting results, making it hard to draw a clear conclusion on clinical implications. Here, we summarize the previous publications on this topic, add new cases of changing MGMT status in relapse and finally combine all reports of more than ten patients in a statistical analysis based on the Wilson score interval. MGMT promoter methylation changes are seen in 115 of 476 analyzed patients (24%; CI: 0.21–0.28). We discuss potential reasons like technical issues, intratumoral heterogeneity and selective pressure of therapy. The clinical implications are still ambiguous and do not yet support a change in clinical practice. However, retesting MGMT methylation might be useful for future treatment decisions and we encourage clinical studies to address this topic.

## 1. Introduction

### 1.1. Epigenetic Changes

The British scientist Conrad Hal Waddington introduced the term “epigenetics” in the early 1940s [1]. Since this time the meaning of “epigenetic changes” has been specified and currently is understood as heritable changes in gene function, without altering the DNA sequence [2,3]. The DNA consists of the four bases adenine, cytosine, guanine, and thymine. After replication, DNA methyltransferases can add a methyl-residue to cytosine, forming 5-methylcytosine [4], often clustered in palindromic CpG dinucleotides in small areas, the so-called CpG-islands throughout the genome [5]. In case such methylated CpG-islands are concentrated in promoter regions, this may lead to silencing of the respective genes [6,7,8,9]. Although DNA methylation-patterns are considered to be quite stable, it has to be noticed, that the methylation profile can change due to external or internal stimuli [7,10]. Indeed, both hyper- as well as hypomethylation do not only play a vital role in long term gene regulation, but also in carcinogenesis by reactivating oncogenes, silencing tumor-suppressor genes, deregulating mRNA expression, promoting mutations or altering the functional stability of chromosomes [4,11].

### 1.2. Glioblastoma Multiforme and Gene Methylation

Methylation patterns play a central role in gliomas. They are not only crucial for the tumor subclassification but might also be targeted by novel treatment modalities [12,13,14]. The current standard in the therapy of glioblastoma multiforme (GBM), the most common primary malignancy of the central nervous system, includes tumor resection, radiation (RT) and concomitant, as well as adjuvant chemotherapy with temozolomide (TMZ) [15]. Although recently the addition of Tumor-Treating Fields (TTFields) to the therapeutic scheme has shown a great potential to improve patients’ outcome, the overall survival (OS) of patients with GBM remains rather unfavorable [16,17,18]. 

Interestingly, in newly diagnosed GBM, methylation of the promoter region of the DNA repair enzyme O^6^-methylguanine-DNA methyltransferase (MGMT) gene has emerged as a strong prognostic factor [19,20,21,22]. MGMT removes alkyl groups from guanine in the DNA, potentially counteracting the therapeutic efficacy of alkylating chemotherapeutics, such as TMZ, in tumor cells [23]. Epigenetic silencing of the promoter region of MGMT might lead to transcriptional repression and a decreased MGMT protein expression [23]. It is associated with an improved response to TMZ chemotherapy and longer overall survival of GBM patients [22,24]. Older patients with MGMT-promoter methylation benefit from TMZ chemotherapy compared to sole radiotherapy, whereas patients without methylation might not [25,26,27,28]. Recently, it has been reported that a combination of the chemotherapeutic lomustine with TMZ was beneficial solely for GBM patients with methylated MGMT promoter, further highlighting the prognostic importance of MGMT promoter methylation status determination [29,30,31]. Nowadays, the patients’ MGMT methylation status is defined by methylation-specific PCR (MSP), high-resolution melting PCR (HRM) or pyrosequencing. A cut-off value of 8–10% has been acknowledged to distinguish unmethylated from methylated MGMT promoters [20,32,33].

However, virtually all GBM tend to relapse, usually associated with increasing aggressiveness and therapy-resistance [34]. Even though the patients’ general condition or the tumor localization often limit the therapeutic approaches in relapse, resection of the recurrent tumor, followed by TMZ-rechallenge is often a viable option [27,28,35]. Therefore, MGMT promoter methylation might not only be predictive for the outcome after primary surgery, but also in tumor recurrence. Nevertheless, its role after disease progression and as a predictor concerning the clinical course after secondary surgery currently remains unclear [24,36].

### 1.3. Rationale and Methodology

**Figure 1:**
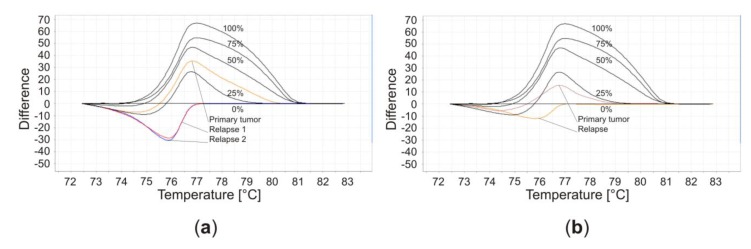
Example of two patients (patient 3 and 7) with a change in O^6^-methylguanine-DNA methyltransferase (MGMT) methylation status as determined by high-resolution melting PCR (HRM). Shown are the melting curves. The methodology has been described in detail elsewhere [37] and is highly accurate compared to methylation-specific PCR (MSP) [55]. (**a**) Patient 3 had a glioblastoma multiforme (GBM) which was methylated between 25% and 50%, while his two relapses were unmethylated. (**b**) Patient 7’s GBM had MGMT promoter methylation between 10% and 25%. His relapse was unmethylated. Patients’ details are summarized in Table 1.

**Figure A1:**
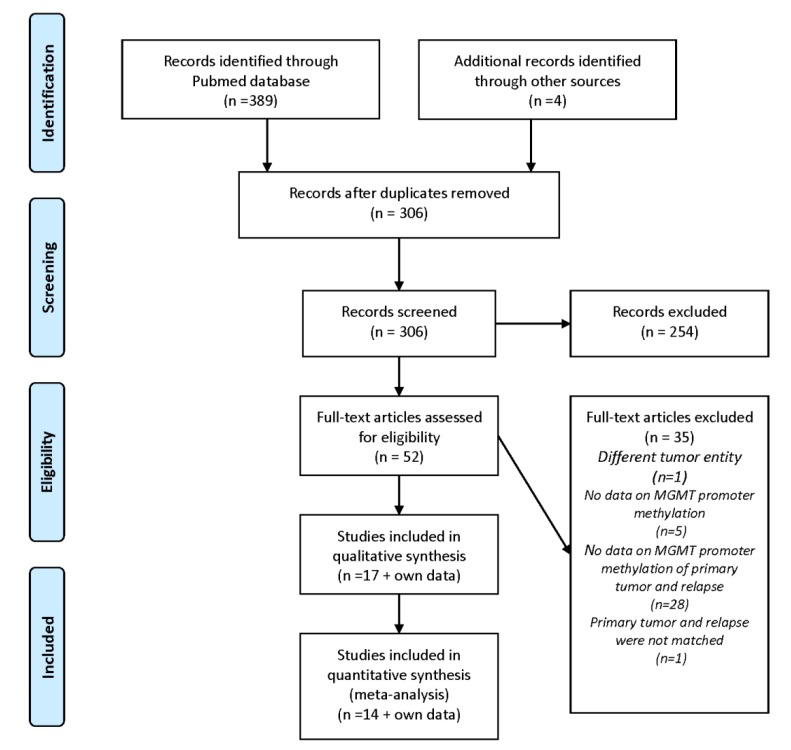
PRISMA guidelines for methodological review of literature related to MGMT promoter methylation changes in glioblastoma relapse.

While working on a study about GBM and their matched recurrences [37], we observed a change of MGMT promoter methylation from primary tumor to relapse in some of the GBM patients. These observations have not been published so far and are summarized in Figure 1 and Table 1. A Pubmed literature search independently conducted by the first and the last author using the keywords “MGMT AND glioblastoma AND relapse”, “O6-methylguanine DNA methyltransferase AND glioblastoma AND relapse” and “MGMT AND methylation AND change” was performed latest on 30 October 2019. The results were limited to English language studies. Articles on different tumor entities, not providing data on MGMT promoter methylation, without information on MGMT promoter methylation of primary tumor and relapse or describing unmatched primary tumor and relapse samples were excluded. A similar screen of the Web of Science database was carried out, but did not yield additional reports of sufficient quality on our topic of MGMT methylation changes in gliomas. This work was conducted in accordance with the PRISMA guidelines (Figure A1). Relevant citations were screened including cross-references and revealed several publications with similar observations as ours. The early data were already reviewed in 2015 [38]. However, in the meantime, several new data have been published, which prompted us to write a new summary of these observations. A couple of them were anecdotal or incidental because they had their main focus on other topics [39,40,41,42,43,44]. Others investigated larger patient cohorts to evaluate, whether the re-determination of the MGMT-methylation status in the relapse situation might be of clinical relevance [36,45,46,47,48,49,50,51,52,53,54]. Different methods for measuring MGMT methylation and diverse selection criteria have been utilized in these reports (Table 2). In addition, some authors came to contrasting conclusions and controversially discussed the underlying mechanisms and clinical significance. Here, we combine these observations, including our own new data, to get a better overview about this interesting and important topic.

## 2. Initial and Incidental Observations of MGMT Promoter Methylation Changes

The first observation of MGMT promoter methylation changes was reported by Komine et al. when evaluating the predictive value of MGMT promoter hypermethylation in patients with low-grade diffuse astrocytomas (LGG) (Table 2) [39]. In a study focusing on the validation of a simplified MSP protocol to determine MGMT promoter hypermethylation, Cankovic et al. compared the primary tumor and relapse of astrocytic tumors of different grading [40]. Precise information about the WHO grading of the tumors or clinical data of the patients is not provided in both studies, due to the distinct main focus of the papers.

Metellus et al. reported an incidental finding of changing methylation status in relapse in GBM patients that underwent surgery and carmustine wafer implantation [41]. However, this change could only be detected by the MethyLight Technique (cut off: >0%) and was not confirmed by MSP. Incidental is also a case report of an epithelial GBM with multiple relapses and methylated MGMT promoter, which changed to a typical, unmethylated GBM [42]. The aim of another study was on the identification of molecular changes following treatment and recurrence of disease with a particular focus on DNA repair pathways [43]. Changes of MGMT promoter methylation were determined by pyrosequencing with a cut-off of 13%. Most recently, differences in miRNA expression between primary tumor and recurrence have been studied [44]. As a side note, the authors also determined MGMT promoter methylation in a subgroup of patients, of which most of the unmethylated samples in primary GBM were methylated in recurrent GBM. Unfortunately, no further subgroup analysis is provided, since the changes in MGMT methylation were not the main topic of this study.

Together, these data imply that the MGMT promoter methylation status is not stable during disease progression and triggered research on potential clinical implications.

## 3. Systematic Clinical Studies on MGMT Promoter Methylation Changes

As the first to address variations of MGMT promoter methylation in GBM, Parkinson et al. performed a small study (Table 2) [45]. Samples were analyzed not only by MSP but also by promoter sequencing, allowing detection of smaller deviations. Gain of methylation was observed in 50% of the patients, loss in 20%, while 30% maintained an unmethylated MGMT promoter status in relapse. Interestingly, these results differ from 2 out of 4 patients gaining methylation when analyzed by MSP. This difference might be due to higher accuracy of promoter sequencing, but also be affected by considering a 4% methylated specimen as positive, while the present cut-off value is at 8–10% [20,32]. It deserves to be noted that the authors describe a patient with a methylated MGMT promoter in primary tumor and recurrence, displaying an increase in the grade of methylation by 28% that could not be detected by MSP.

Christmann et al. examined the interrelation between the MGMT methylation status and MGMT activity in GBM patients [46]. Generally, their data show an overall correlation between promoter methylation and lacking or low MGMT activity. They also compared primary GBM samples with their matched recurrences. It was found that 22% were initially methylated with low MGMT activity. One of these tumors displayed reduced and the other one lost MGMT promoter methylation during the clinical course, with maintaining and increasing MGMT activity, respectively. Another study, evaluating variations of MGMT promoter methylation and protein expression after adjuvant treatment in GBM patients came to very similar results [47]. Generally, there was a correlation between the methylation status of the MGMT promoter and MGMT protein expression in 65% of the analyzed cases. It was found that 33% of recurrent GBM had changed their MGMT promoter methylation compared directly to the matched primary GBM from the same patient. A direct, short-term influence of the therapy on MGMT promoter methylation could not be shown in a cell culture experiment. A Japanese research group observed correlation of MGMT mRNA expression with MGMT promoter methylation [54]. They analyzed astrocytic tumors WHO grade II–IV, of which 83% lost their initial methylation, while the umethylated tumors did not change in relapse. The authors speculate that therapy resistant clones or glioma stem cells may survive to form recurrent tumors.

In a larger study, 37% GBM patients treated with RT and TMZ chemotherapy changed the MGMT methylation status in relapse [48]. In 2017, these authors extended their study and found the MGMT methylation status to be stable in 75% of the patients [36]. However, seen from another perspective, it means that 25% changed their MGMT promoter methylation status. Only patients adjuvantly treated with RT and TMZ were included to avoid a potential bias caused by the impact of different post-surgical treatments on genetic and epigenetic alterations. Both studies revealed that OS was significantly correlated with the MGMT methylation status of the primary tumor, but not at relapse, therefore showing no implication towards repetitive MGMT methylation testing in tumor recurrence.

In contrast to previous observations, Felsberg et al. reported a relatively low percentage of 11% MGMT promoter methylation changes in relapse, of which they say only 6% can be considered as true changes, since their loss or decrease of MGMT promoter methylation at tumor recurrence could not be explained by a low tumor cell content of the respective tissue samples [49]. They conclude that the MGMT promoter methylation status does not change from primary to recurrent tumor in the vast majority of GBM patients and due to this fact they see no implication for repetitive testing in daily clinical routine.

Similarly, Park et al. detected MGMT promoter methylation changes in only 8%, as detected by MSP [50]. However, when analyzing 12 of these patients by methylation-specific multiplex ligation probe amplification (MS-MLPA), they observed a decrease in the MGMT promoter methylation ratio in 75% of the samples). They conclude that such alterations frequently remain undetected in case only MSP is utilized.

O’Regan et al. used pyrosequencing with a cut-off value of 9% for their analysis [51]. While 64% of the patients remained stable, 36% changed their methylation status. They hypothesize that such changes might be related to TMZ treatment. Although these authors did not find a significant influence of the methylation changes on progression free survival (PFS) or OS of the patients, they highlight the need to reappraise the methylation status post-treatment.

Another study reports a change of the MGMT promoter methylation status in 27% [52]. This study is distinctive to the other reports, as the authors took several samples from each individual tumor, one from the periphery and another one from the central part. In 80% of all cases the MGMT methylation was homogenous throughout the tumor. However, in 20% of the tumors the peripheral part was unmethylated, while the central part was methylated and only these initially heterogeneously methylated tumors changed their methylation status in relapse. Therefore, the authors speculate that this change might be due to clonal expansion of formerly heterogenous tumor cells leading to homogenous tumor recurrence.

The most recent publication on this topic reports patients with astrocytic tumors of different WHO grading [53]. Thus, 12 changes in MGMT promoter methylation status could be observed by MS-MLPA. However, none of them occured in GBM. Mostly affected were WHO grade III gliomas, especially in relation with adjuvant RT, but not with irradiation in combination with chemotherapy.

Finally, we would like to add our own GBM patients to these studies (Table 1, Table 2). Primary tumors and the matched relapses were analyzed by HRM with a cut-off value of 10%. It could be seen that 38% had a change in MGMT promoter methylation. Switches occurred into both directions. In addition to the global changes described above, HRM allows to detect differences in the grade of methylation. Three patients with a positive methylation status in both primary tumor and relapse increased their MGMT promoter methylation by at least 20%. Therefore, a total of 62% displayed unstable methylation. Three patients presented with a second relapse. However, although in one patient the primary tumor was methylated, both of his relapses were unmethylated (Figure 1). The other patients had a methylated primary tumor and maintained this status in both relapses. We did not detect any correlation of the MGMT promoter methylation status in relapse with the OS of the patients. Therefore, our data are consistent with the observations of several authors reviewed above in that we do not see a justified clinical implication to re-evaluate the MGMT promoter methylation in relapse, yet.

## 4. Combined-Analysis

**Figure 2:**
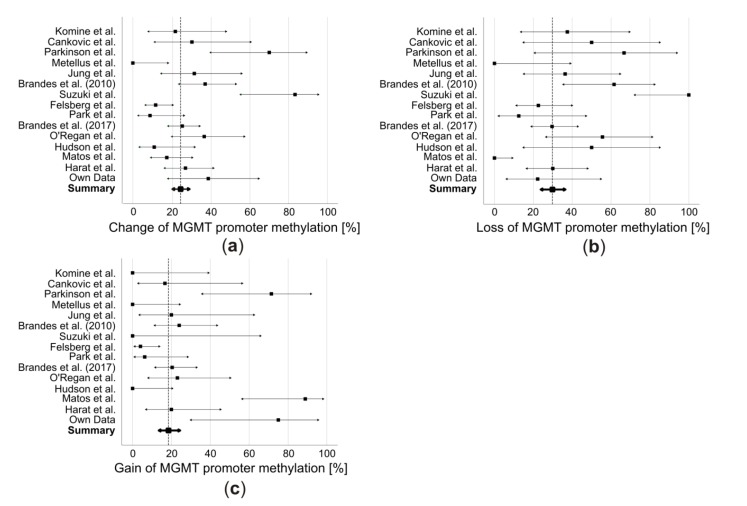
Combined analysis of selected data from publications reporting changes in MGMT promoter methylation in GBM relapse using IBM SPSS Statistics 25 (IBM, New York, NY, USA). For this analysis only studies investigating at least 10 patients were included. In addition, changes were analyzed based on the cut-off value of the respective study and not based on the degree of methylation. In case MGMT promoter methylation was reported to be analyzed by two or more different methods, we focused on the method with the larger number of patients. Only patients with a reported MGMT status in the primary tumor and matching relapse were included. Therefore, these data often have been extracted from tables or supplementary material published with the respective paper. (**a**) Changes of MGMT promoter methylation in both directions. (**b**) Loss and (**c**) gain of MGMT promoter methylation. Black squares: percentage of patients with change, loss or gain of MGMT promoter methylation; arrows: 95% confidence interval (CI, calculated based on the Wilson score interval, [57]); vertical dashed line: overall percentage of MGMT promoter methylation changes from the combined analysis of the listed publications.

The main drawback of most of the above-mentioned studies is their small sample size. However, if considering all those publications that describe at least 10 patients, together they comprise 476 patients. In a combined analysis, 115 patients (24%; CI: 0.21–0.28) changed their MGMT promoter methylation during the clinical course (Figure 2a). The changes divide into 71 patients (30%; CI: 0.24–0.36) switching from methylated to unmethylated (Figure 2b) and 44 patients (18%; CI: 0.14–0.24) converting from unmethylated to methylated (Figure 2c).

Main limitations of such a combined analysis are the inhomogeneity of cut-off values, selection criteria, utilized methods, and a potential positive publication bias. Additionally, 4 of the included studies analyzed not only GBM but also LGG [39,40,53,54]. Therefore, the limitation of our analysis derives from such limitations of the original studies.

## 5. Potential Reasons for MGMT Promoter Methylation Changes and Their Clinical Implications

Technical issues, intratumoral heterogeneity and selective pressure of cytotoxic agents are discussed as reasons for the observed changes in MGMT promoter methylation during the clinical course [36].

MSP is the most widely used method, followed by pyrosequencing, MS-MLPA, MethyLight technique and HRM (Table 2) [20]. While MSP allows a methylated/unmethylated classification, the other methods provide additional information, e.g., quantification of the grade of promoter methylation and its percentual increase or decrease [20]. However, while all these methods are established and usually of high accuracy [55], sample quality might be an issue [49]. For example, a false negative determination of MGMT promoter methylation by MSP due to necrotic tissue within the sample has been reported [58]. Contamination of the tumor tissue with normal and/or inflammatory cells also might lead to an incorrect estimation of the grade of MGMT promoter methylation. Felsberg et al. state that approximately half of their observed changes were mainly due to low tumor cell content in one of the samples [49]. This questions some of the reviewed data without available information about the tumor cell quantity in the analyzed samples. However, only larger discrepancies seem to influence the measurement, as tumors differing in tumor cell content up to 40% are stated to give comparable results [59]. Therefore, the reported changes might be explained by technical inaccuracies only partially, while the majority are not.

MGMT promoter methylation usually is seen as homogenous within the tumor [45,58,60], although some groups reported contrasting results [21,61]. Up to 33% of the GBM were described to be heterogeneous with different MGMT promoter methylation status in the intermediate part of the tumor compared to the peripheral and inner parts [61]. This finding is in line with the observations by Barresi et al., characterizing 20% of the GBM to be unmethylated at the peripheral parts of the tumor, but methylated at the center and only these heterogeneous tumors were the ones changing their methylation status in relapse [52]. Therefore, intra-tumoral heterogeneity as a partial reason for MGMT promoter methylation changes cannot be excluded and therapy-induced eradication of sensitive tumor clones and clonal expansion of resistant ones may be involved in this phenomenon.

Interestingly, MGMT promoter methylation might not only be predictive for the TMZ-response but also altered by it. TMZ treatment may lead to DNA hypermethylation followed by global demethylation, raising the question whether TMZ might induce resistance against itself by reshaping the methylation pattern [62,63]. Again, the results of the different studies are contradictory. While some did not see a correlation between TMZ treatment and a change of MGMT promoter methylation [47,49], others drew a different conclusion [48,64]. The subgroup of patients with methylated MGMT promoter in the primary tumor displayed a significant loss of methylation if treated with concurrent RT/TMZ (7 of 8) compared to patients treated with RT followed by sequential TMZ (1 of 5, *p* = 0.03). Accordingly, the authors discuss whether the type of post-surgical treatment may influence the MGMT methylation status [48]. We attempted to perform a subgroup meta-analysis comparing patients initially treated with RT only versus patients treated with concomitant radiochemotherapy. However, our analysis revealed that the number of patients treated with RT only was insufficient and mainly reported by older studies. Therefore, we refrained from presenting these data. However, Felsberg et al. tested this hypothesis. Their comparison between patients treated with TMZ and RT versus RT alone showed no significant differences in changes of the MGMT promoter status, indicating that the observed distinctions were not affected by TMZ treatment [49]. Further, their data revealed a correlation between MGMT promoter methylation and OS, as well as MGMT promoter methylation at second surgery and post-recurrence survival, diverging from the observations of Brandes et al., who detected a significant correlation of MGMT methylation at first and second surgery with OS, but not at second surgery with post-recurrence survival [36,48]. The observed changes were almost equally distributed to losses (*n* = 16) and gains (*n* = 11) of MGMT promoter methylation, suggesting TMZ to possibly not be causing a significant selection pressure. This view is shared by Storey et al., who recently proposed a mathematical model to predict GBM response to TMZ treatment [64]. They conclude that the changes in methylation cannot be explained by evolutionary selection alone, but might indeed occur due to TMZ altering the cells’ general methylation profile. Although a final conclusion on this topic cannot be drawn, several groups agree that there is currently no definitive implication for clinical retesting of the MGMT promoter methylation status in the relapse situation [36,49].

## 6. Conclusions

There are clear indications that the MGMT promoter methylation can change from the primary tumor to relapse in about 24% of glioma patients. The possible causes, molecular mechanisms, and clinical impacts of these alterations remain ambiguous, with sometimes contrasting results in the literature. Recent observations indicate that alterations of the cells’ general methylation profile by TMZ might be one important reason for changes of the MGMT promoter methylation status in recurrence. This should be further investigated. While there is currently no definitive implication for changing the standard of practice or guidelines, our present state of knowledge seems too restricted to draw final conclusions. Testing MGMT promoter methylation not only in the primary tumor, but also in relapse might be useful. We highly recommend that clinical studies on GBM recurrence acknowledge MGMT promoter methylation changes and investigate whether these changes should be considered in the treatment decision.

## Figures and Tables

**Table 1:** Summary of patients’ clinical data.

	Patient Characteristics			Tumor Characteristics						Therapy			Outcome		
Patient	Sex	Age [years]	ECOG	Tumor Volume [ccm]	Tumor Localization [hemisphere/lobe]	IDH Mutation	MGMT Promoter Methylation		Ki67 Staining [%]	Extent of Resection	RT	TMZ	Relapse	OS [months]	PFS [months]
							Primary Tumor	Relapse							
1	Female	69	1	33.6	Right/occipital	No	Negative	Negative	20	Subtotal	Yes	Yes	Local	18	3
2	Female	49	1	25.5	Left/parietal	No	Positive	Positive	35	Subtotal	Yes	Yes	Local	31	15
3	Male	42	0	54.1	Left/parietal	No	Positive	Negative	30	Subtotal	Yes	Yes	Local	45	12
4	Female	70	1	9.3	Left/temporal	No	Positive	Positive	10	Subtotal	Yes	Yes	Local	30	3
5	Female	59	0	8.2	Left/temporal	No	Positive	Positive	20	Subtotal	Yes	Yes	Local	14	4
6	Male	48	0	58.2	Right/frontal	No	Positive	Positive	30	Total	No	Yes	Local	34	22
7	Male	66	1	19.1	Left/frontal	No	Negative	Positive	20	Subtotal	Yes	Yes	Local	15	4
8	Male	58	3	89.9	Right/occipital	No	Positive	Positive	25	Subtotal	Yes	Yes	Multifocal	24	4
9	Male	49	0	54.8	Left/frontal	Yes	Negative	Positive	30	Total	Yes	Yes	Local	48	6
10	Male	60	1	79.2	Left/temporal	Yes	Positive	Negative	25	Total	Yes	Yes	Local	25	7
11	Male	22	3	2.2	Left/frontal	Yes	Positive	Positive	35	Subtotal	Yes	Yes	Multifocal	27	10
12	Female	47	1	2.3	Left/frontal	No	Positive	Positive	25	Subtotal	Yes	Yes	Multifocal	47	7
13	Male	74	0	60.7	Left/temporal	No	Negative	Positive	15	Subtotal	Yes	No	Local	12	7
**Total**	**Female:** 5/38.5% **Male:** 8/61.5%	**Median:** 58	**Median:** 1	**Median:** 33.6	**Left:** 10/76.9% **Right:** 3/23.1% **Frontal:** 5/38.5% **Temporal:** 4/30.8% **Occipital:** 2/15.4% **Parietal:** 2/15.4%	**No:** 10/76.9% **Yes:** 3/32.1%	**Negative:** 4/30.8% **Positive:** 9/69.2%	**Negative:** 3/23.1% **Positive:** 10/76.9%	**Median:** 25	**Total:** 3/23.1% **Subtotal:** 10/76.9%	**Yes:** 12/92.3% **No:** 1/7.7%	**Yes:** 12/92.3% **No:** 1/7.7%	**Local:** 10/76.9% **Multifocal:** 3/23.1%	**Median:** 27	**Median:** 7

MGMT promoter methylation was determined by high-resolution melting PCR (HRM) with a cut-off value of 10% [37]. Following the recommendations of the World Health Organisation [56], immunohistochemical IDH1 R132H negative GBM of patients below the age of 55 years were pyrosequenced for IDH1 and IDH2 mutations. If not stated otherwise, all information relate to the time of diagnosis. ECOG: performance status of the Eastern Cooperative Oncology Group; IDH: Isocitratdehydrogenase; MGMT: O^6^-methylguanine DNA methyltransferase; OS: overall survival; PFS: progression-free survival; TMZ: temozolomide.

**Table 2:** Summary of publications presenting GBM patients with changes in MGMT promoter methylation in relapse.

	Patients’ Characteristics					MGMT-Status					Therapy			Outcome		
Authors	Tumorgrade	Total Patients	Male	Female	Median Age at Diagnosis [years]	Methylated Primary Tumor	Unmethylated Primary Tumor	Methylated Relapse	Unmethylated Relapse	MGMT Status in Relapse (Identical/Loss/Gain)	Extent of Tumor Resection (Total/Subtotal/Biopsy)	RT (RT Only/ Concomitant RT + TMZ)	Adjuvant TMZ Therapy	Median PFS [months]	Median OS [months]	Method and (Type of Tissue)
Komine et al. (2003)	LGG	14				8	6	11	3	11/3/0						MSP (PE)
Cankovic et al. (2007)	LGG GBM	10				4	6	3	7	7/2/1						MSP (PE)
Parkinson et al. (2007)	GBM	10	8	2	55	3	7	6	4	3/2/5		8/0	9			Promoter sequencing & MSP (FF)
Metellus et al. (2009) ^1^	GBM	18				6	12	6	12	18/0/0						MSP & methyLight technique (FF)
Christmann et al. (2010) ^2,3^	LGG GBM	9				2/0	7	1/1	8	7/2/0						MSP (PE)
Jung et al. (2010)	GBM	16	7	9	53	11	5	9	7	11/4/1	15/1/0	14/2	16		19.5	MSP (PE)
Brandes et al. (2010)	GBM	38	28	10	49	13	25	11	27	24/8/6	27/10/1	11/27	38	12.0	24.3	MSP (PE)
Suzuki et al. (2010)	LGG GBM	12	9	3	52	10	2	0	12	2/10/0		1/5	5	18.0		MSP (PE & FF)
Tanaka et al. (2010) ^2^	GBM	1	1	0	55	1	0	0	1	0/1/0	1/0/0	1/0	0			MSP
Felsberg et al. (2011) ^3^	GBM	80	58	22	57	31/3	49	26/3	54	71/7/2	40/33/6	16/64	56	9.1	18.3	MSP & Pyro- sequencing (FF)
Park et al. (2012)	GBM	24	15	9	60	8	16	8	16	22/1/1		9/14	24	8.0		MSP & MS-MLPA (PE)
Brandes et al. (2017)	GBM	108	69	39	51	54	54	49	59	81/16/11	50/42/16	0/108			24.4	MSP & Pyro- sequencing (PE)
O’Regan et al. (2017)	GBM	22	12	10	50	9	13	7	15	14/5/3		0/22	22			Pyrosequencing (PE)
Hudson et al. (2018)	GBM	19	12	7	60	4	15	2	17	17/2/0		0/19	19	7.0	15.0	Pyrosequencing (FF)
Matos et al. (2018) ^4^	GBM	47				38	9	46	1	39/0/8			47			MSP (PE)
Barresi et al. (2018) ^5^	GBM	11	4	7	60	4	10	3	8	11/0/0	11/0/0	0/11	11	16.0	27.0	MSP (PE)
Harat et al. (2019) ^6^	LGG GBM	45			45	30	15	27	18	33/9/3		14/20				MS-MLPA (PE)
Own Data	GBM	13	8	5	58	9	4	10	3	8/2/3	3/10/0	1/11	12	7.0	27.0	HRM (FF)
**Summary** ^5^		**485**				**238**	**253**	**229**	**260**	**377/64/44**						

If a study used 2 different methods for determining MGMT promoter methylation, only the method with data on more patients was considered in the combined analysis (Figure 2). ^1^ This study detected one change by MethyLight technique and none by MSP. ^2^ These studies were excluded from the combined analysis due to the patient number being below 10. ^3^ These studies divided positive promoter methylation status into two groups: all positive tumors vs. weakly positive or hemimethylated. ^4^ This study only provides the number of patients with vs. without methylation at diagnosis and relapse, but does not provide information on individual changes. Therefore, we included the minimal number of changes that must have occurred. ^5^ In Barresi et al. tumor heterogeneity was analyzed, reporting 3 of the primary tumor samples to be methylated in the center and unmethylated in the periphery. Therefore, they do not describe a “real change”, but rather a subclonal expansion in tumor recurrence. These tumors were counted as methylated and unmethylated alike. Consequently, total numbers do not add up and this study was excluded from the combined analysis. ^6^ This study distinguished 4 different methylation groups; however, we converted this information into “methylated” and “unmethylated”. FF: fresh frozen tissue; GBM: glioblastoma multiforme; HRM: high-resolution melting PCR; LGG: low grade glioma WHO grade I-III; MGMT: O^6^-methylguanine DNA methyltransferase; MS-MLPA: methylation specific multiplex ligation-dependent probe amplification; MSP: methylation-specific PCR; OS: overall survival; PE: paraffin-embedded tissue; PFS: progression free survival; RT: radiotherapy; TMZ: temozolomide.
